# Dihydroartemisinin Sensitizes Mutant p53 (R248Q)-Expressing Hepatocellular Carcinoma Cells to Doxorubicin by Inhibiting P-gp Expression

**DOI:** 10.1155/2019/8207056

**Published:** 2019-12-31

**Authors:** Yue Yang, Jianxin He, Jing Chen, Li Lin, Yongqi Liu, Cunmin Zhou, Yun Su, Hulai Wei

**Affiliations:** ^1^Key Laboratory of Preclinical Study for New Drugs of Gansu Province, School of Basic Medical Sciences, Lanzhou University, Lanzhou 730000, Gansu, China; ^2^Key Laboratory of Pharmacology and Toxicology of Traditional Chinese Medicine of Gansu Province, Gansu University of Chinese Medicine, Lanzhou 730000, Gansu, China

## Abstract

Mutant p53 (R248Q) induces doxorubicin (ADM) resistance in hepatocellular carcinoma (HCC). Dihydroartemisinin (DHA) can synergistically enhance anticancer effect of many chemotherapeutic agents. However, whether DHA could increase therapeutic efficacy of ADM in p53 (R248Q)-expressing HCC cells remains unknown. In the present study, we established mutant p53 (R248Q)-expressing Hep3B cells to study the effect and mechanism of DHA on ADM resistance and the synergistic effect of DHA with ADM. We found that P-gp was highly expressed in p53 (R248Q)-expressing Hep3B cells. As a result, cells expressing p53 (R248Q) displayed higher cell viability and lower cell apoptosis level upon ADM treatment. Meanwhile, phosphorylation levels of ERK1/2 and p65 were elevated in p53 (R248Q)-expressing Hep3B cells. However, combination of DHA and ADM treatment decreased cell viability and elevated cell apoptosis level in p53 (R248Q)-expressing Hep3B cells. Molecular dynamics simulations showed that DHA had the potential to bind with mutant p53 (R248Q) protein. Furthermore, DHA treatment decreased P-gp expression and inhibited phosphorylation levels of ERK1/2 and p65 in p53 (R248Q)-expressing Hep3B cells. Finally, DHA treatment could significantly reduce ADM efflux in p53 (R248Q)-expressing cells. Our results indicate that DHA could decrease P-gp expression via inhibiting the p53 (R248Q)-ERK1/2-NF-*κ*B signaling pathway, which eventually confers sensitization of p53 (R248Q)-expressing HCC cells to ADM. Our study provides evidence for the potential application of DHA and ADM combination in treatment of mutant p53 (R248Q)-harbored HCC.

## 1. Introduction

Liver cancer is currently the third leading cause of cancer-related mortality worldwide [1, 2]. Hepatocellular carcinoma (HCC) is the most common type of liver cancer [3]. Chemotherapy is a major treatment for HCC patients because most patients are diagnosed at advanced stages when the surgical excision is infeasible [4]. Doxorubicin (ADM) is a commonly used drug for HCC [5]. However, long-term use of ADM inevitably causes drug resistance and greatly reduces the treatment efficacy in HCC patients [6].

P-glycoprotein (P-gp), encoded by multidrug resistance gene *MDR-1*, is an important target for drug resistance in many cancers [7, 8]. P-gp belongs to the family of ATP-dependent membrane pumps, which can facilitate the efflux of chemotherapeutic drugs such as ADM, vincristine, and paclitaxel, thus resulting in drug resistance [9–11].

Tumor suppressor protein p53 accumulates in the nucleus to induce the expression of genes related with cell cycle arrest and apoptosis upon cell stress [12]. It is worth to note that *TP53* gene is frequently mutated in more than 50% human cancers [13]. Some p53 mutants acquire additional functions, called gain-of-functions (GOFs), which confer new properties to p53 [14]. One of the GOFs is to induce the expression of P-gp, which further induces resistance to chemotherapeutics [15, 16]. The frequent mutations in *TP53* gene are missense mutations in the DNA-binding domain, including R175H, R248Q, R249S, and R273H [17]. Among them, R248Q is the most frequent mutation in HCC and the second frequent mutation in other human cancers [18, 19]. Likewise, p53 (R248Q) mutation acquires a novel GOF, which is to induce the expression of P-gp [18]. Thus, development of new agents which may inhibit p53 (R248Q)-mediated P-gp expression is desirable for the treatment of resistant HCC.

Dihydroartemisinin (DHA), one of the most active derivatives of artemisinin, is originally used as an antimalarial agent [20]. Recently, DHA has been found to exhibit potent anticancer properties in different kinds of human HCC cells [21, 22]. Meanwhile, DHA induces apoptosis in human HCC cells harboring p53-null, wild-type (WT) p53, and mutant p53, respectively [23]. Furthermore, the combination of DHA and other chemotherapeutic agents plays a synergistic role in the treatment of many kinds of cancers [24, 25]. However, whether DHA could enhance the sensitivity of p53 (R248Q)-expressing HCC cells to ADM and the underlying mechanism remains unknown.

In this study, we examined the effect of DHA on ADM resistance in mutant p53 (R248Q)-expressing HCC cells and the synergistic effects of DHA and ADM combination in mutant p53 (R248Q)-expressing HCC cells. The underlying mechanisms were analyzed and discussed. We found that the combination of DHA with ADM significantly reduced the cell viability and induced apoptosis of p53 (R248Q)-expressing HCC cells, indicating the synergistic effects of DHA and ADM. We further demonstrated that DHA decreased the expression of P-gp via inhibiting p53 (R248Q)-ERK1/2-NF-*κ*B signaling, which leads to the reversal of ADM resistance in p53 (R248Q)-expressing HCC cells. Our study provides experimental evidence for the use of DHA and ADM combination in treating HCC harboring mutant p53 (R248Q).

## 2. Materials and Methods

### 2.1. Cell Line and Cell Culture

Hep3B (Cell Bank, Shanghai Institutes for Biological Sciences of the Chinese Academy of Sciences, China), which is a p53-null human HCC cell line, was cultured in MEM with L-glutamine (Corning, USA) supplemented with 10% FBS (MRC, Uruguay) and incubated at 37°C in humidified atmosphere with 5% CO_2_.

### 2.2. Establishment of p53-Expressing Cells

PLX304 vector containing wild-type p53 cDNA was purchased from DNASU (USA). R248Q mutation was generated from WT p53 cDNA by using the site-directed mutagenesis method. The primer sequences were R248Q primer (F) ATGGGCGGCATGAACCAGAGGCCCATCCTCACC and R248Q primer (R) GGTGAGGATGGGCCTCTGGTTCATGCCGCCCAT. The lentiviral vectors containing WT and R248QcDNAs were constructed through inserting the cDNAs into PLVX-puro vectors. Lenti-virus expression systems including PLVX-puro-p53 cDNA, PMD2.G, and PSPAX2 were transfected into HEK293T cells using FuGENE 6 Reagent (Promega, USA). Transfected HEK293T cells were cultured for another 48 h. The culture supernatant was collected and filtered with 0.22 *μ*m filter to isolate the lentiviruses. Then, the lentiviruses were used to infect Hep3B cells for 48 h. The infected Hep3B clones with stable expression of p53 WT and p53 R248Q were screened in a medium supplemented with 4 *μ*g/mL puromycin (Sigma-Aldrich, USA) for another 10 d. The stable clones were used for subsequent experiments.

### 2.3. MTT Assay

Empty vector, WT p53 or p53 (R248Q)-expressing Hep3B cells (5 × 10^3^) were seeded in a 96-well plate and incubated for 24 h, respectively, followed by 0.4, 0.8, 1.6, 3.2, and 6.4 *μ*M ADM treatment (TRC, Canada) for 48 h. Additionally, 5 × 10^3^ p53 (R248Q)-expressing Hep3B cells were seeded in a 96-well plate and pretreated with 5 *μ*M DHA (CCGB, China) for 24 h, followed by 2 *μ*M ADM (TRC, Canada) treatment for another 48 h. Then, 0.5 mg/mL MTT (Solarbio, China) was added into each well for 4 h at 37°C. Formazan crystals in viable cells were solubilized with DMSO. The absorbance at 490 nm was recorded with the Microplate Reader (Victor X, PerkinElmer).

### 2.4. Clone Formation Assay

Empty vector, WT p53 or p53 (R248Q)-expressing Hep3B cells (2 × 10^3^) were seeded in six-well plates and incubated for 24 h. Additionally, p53 (R248Q)-expressing Hep3B cells (2 × 10^3^) were seeded in a 96-well plate and pretreated with 5 *μ*M DHA for 24 h. Then, the cells in each group were treated with 2 *μ*M ADM for 24 h, followed by culturing in a fresh culture medium for another 14 d. The culture medium was changed every 3 d. At the end of the treatment, the cells were fixed with 4% paraformaldehyde (BioFroxx, Germany) and stained with crystal violet staining solution (Solarbio, China). The colony number was counted.

### 2.5. Cell Morphology

Empty vector, WT p53 or p53 (R248Q)-expressing Hep3B cells (2 × 10^3^) were treated with 2 *μ*M ADM for 24 h. Additionally, p53 (R248Q)-expressing Hep3B cells (2 × 10^3^) were pretreated with 5 *μ*M DHA for 24 h, followed by 2 *μ*M ADM for another 24 h. Then, cells were stained with Giemsa–Wright stain (Solarbio, China). The cells in each group were observed under AX80 optical microscopy (Olympus, Japan).

### 2.6. Western Blot

Cells were lysed in 200 *μ*L RIPA lysis buffer (Solarbio, China). The lysates were centrifuged at 12,000 rpm for 7 min at 4°C. The concentration of proteins was measured by a BCA assay kit (Thermo, USA) according to the instruction. Total proteins of 5 *μ*g were separated by SDS-PAGE (12% acrylamide) and transferred to poly vinylidenedifluoride membranes (Millipore, USA). The membranes were incubated in 5% skim milk for 2 h at room temperature and subsequently with primary antibodies (1 : 1000 dilution) overnight at 4°C. The primary antibodies included rabbit polyclonal anti-P-gp, p53, phosphor-ERK1/2 (Thr202/Tyr204), ERK1/2, phosphor-AKT (Ser473), AKT, phosphor-p65 (Ser536), p65, and rabbit polyclonal anti-GAPDH antibody, which were all from Cell Signaling Technology (USA). After washing, the membranes were incubated with peroxidase-AffiniPure goat anti-rabbit IgG (H + L) (ZSGBBio, China) for 2 h. After washing again, the bands were visualized with enhanced chemiluminescence reagents (Millipore, USA).

### 2.7. Cell Apoptosis Analysis

Empty vector, WT p53 or p53 (R248Q)-expressing Hep3B cells (1 × 10^5^) were treated with 2 *μ*M ADM for 24 h. Additionally, p53 (R248Q)-expressing Hep3B cells (1 × 10^5^) were pretreated with 5 *μ*M DHA for 24 h, followed by 2 *μ*M ADM for another 24 h. Cells were collected and washed with cold PBS, stained with binding buffer containing 4 *μ*L Annexin V-FITC and 2 *μ*L propidium (PI) (Bestbio, China), and followed by detecting with flow cytometry (LSRFortessa, BD Biosciences, USA).

### 2.8. Cellular Uptake and Retention of ADM

For cellular uptake detection, 1 × 10^5^ cells were cultured in 12-well plates for 24 h and treated with 2 *μ*M ADM for 6 h. Cells were collected and resuspended in 400 *μ*L PBS to measure fluorescence intensity of ADM via flow cytometry (LSRFortessa, BD Biosciences, USA) (excitation at 488 nm and emission at 575 nm).For retention detection, 1 × 10^5^ cells were cultured in 12-well plates for 24 h and treated with 2 *μ*M ADM for 6 h. After that, the culture medium was replaced with ADM-free medium, and the cells were cultured for additional 2 h and 4 h, respectively. Cells were collected and resuspended in 400 *μ*L PBS to measure fluorescence intensity of ADM via flow cytometry (LSRFortessa, BD Biosciences, USA) (excitation at 488 nm and emission at 575 nm).

### 2.9. Molecular Dynamics Simulations

We used Schrodinger's kit to study the docking potential of DHA with mutant p53 (R248Q) protein. The crystal structure of WT p53 protein was obtained from PDB database (PDB ID : 4HJE). The structure is a tetramer consisting of homologous peptide chains, all of which were retained in subsequent docking. The original crystal structure was prepared before docking using the protein processing function in the Schrodinger Maestro module. The process included hydrogenation, filling the missing residues, and adjusting the whole protonation state of the protein to meet the condition of pH = 7.0. Finally, OPLS3 force field was used to optimize the position of hydrogen atom in protein. On the basis of WT p53 structure, we mutated ARG248 into GLN248 (R248Q) in four chains. After obtaining the abovementioned structure, we produced docking sites for p53 (R248Q) protein. The center of the box used in the docking process was located at the center of residue 248 in chain A. The side length of the box was 25 Å. There was no rotatable hydrogen atom in the docking process. Subsequently, the docking work was completed by the Virtual Screening Module (VSW) in the Maestro module. The small molecule used for docking was DHA, which produced 10 stereoisomers for the unspecified stereocenter and 10 minimum energy structures for the ring structure. All conformations generated by docking were retained, and the lowest energy conformations were scored by MM/GBSA.

### 2.10. Statistical Analysis

SPSS13.0 was used for data analysis. The data were shown as means ± standard deviations (SD) of three independent assays. Statistical analysis was carried out by using one-way ANOVA. *p* values of *p* < 0.05 were considered to indicate statistically significant differences.

## 3. Results

### 3.1. Mutant p53 (R248Q) Induces P-gp Expression and ADM Resistance in Hep3B Cells

**Figure 1:**
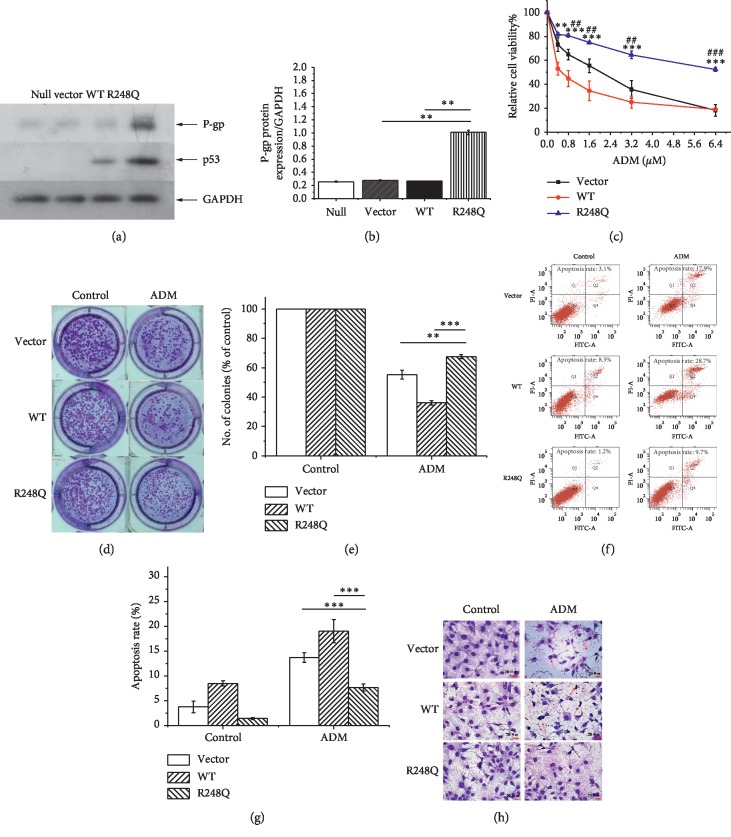
p53 (R248Q) induced P-gp expression and ADM resistance in Hep3B cells. (a) Western blot analysis for the expression of p53 and P-gp in Hep3B-derived clones. Null: Hep3B, Vector: empty vector lenti-virus-infected Hep3B cells, WT : Hep3B cells expressing WT p53, and R248Q : Hep3B cells expressing p53 (R248Q). (b) Quantitative analysis of P-gp in Hep3B-derived clones. (c) Detection of cell viability upon ADM treatment by MTT assays. (d) Representative image of colony formation. (e) Quantitative graph of colony numbers. (f) Detection of cell apoptosis by flow cytometry. (g) Quantitative graph of apoptotic rates. (h) Morphological changes of cells (magnification ×400). The red arrowhead indicated membrane invagination, nuclear condensation, and vacolation in apoptotic cells. The data represent the mean ± SD of 3 independent experiments. For (c), ^*∗∗*^*p* < 0.01 and ^*∗∗∗*^*p* < 0.001 compared with the WT p53 group. ^#^*p<0*.05, ^##^*p* < 0.01, and ^###^*p* < 0.001 compared with the control group. For (b), (e), and (g), ^*∗∗*^*p* < 0.01 and ^*∗∗∗*^*p* < 0.001.

In order to analyze the effects of p53 (R248Q) on P-gp expression, we firstly constructed Hep3B cells expressing mutant p53 (R248Q) and detected P-gp expression in Hep3B-derived cells. p53 (R248Q)-expressing cells showed obviously increased P-gp expression compared with either empty vector lenti-virus infected cells (control cells) or WT p53-expressing cells (Figures 1(a), 1(b)). Then, we examined the ADM resistance in p53-expressing cells by detecting cell survival after ADM treatment. The results showed that cells expressing p53 (R248Q) exhibited significantly higher survival in comparison with either control cells or WT p53-expressing cells upon ADM treatment (Figure 1(c)). Furthermore, colony formation assay showed that clone numbers in p53 (R248Q)-expressing cells were significantly more than those in either control cells or WT p53-expressing cells upon ADM treatment (Figures 1(d) and 1(e)). Cell apoptosis analysis results showed that ADM treatment for 24 h could induce apoptosis in cells expressing WT p53 and control cells obviously, while a more poor extent of apoptosis in cells expressing p53 (R248Q) (Figure 1(f)). The statistical results showed that ADM-induced cell apoptotic level in p53 (R248Q)-expressing cells was significantly lower than that in either empty vector or WT p53-expressing cells (*p* < 0.01) (Figure 1(g)). We subsequently performed cell morphology observation, and the results revealed that empty vector and WT p53-expressing cells showed more apparent apoptotic phenotype in response to ADM treatment, such as membrane invagination, nuclear condensation, and vacolation, compared with that in p53 (R248Q)-expressing cells (Figure 1(h)). The above results indicate that mutant p53 (R248Q) induced P-gp expression and ADM resistance in Hep3B cells.

### 3.2. DHA Synergistically Enhances Apoptosis-Inducing Effect of ADM in p53 (R248Q)-Expressing Hep3B Cells

**Figure 2:**
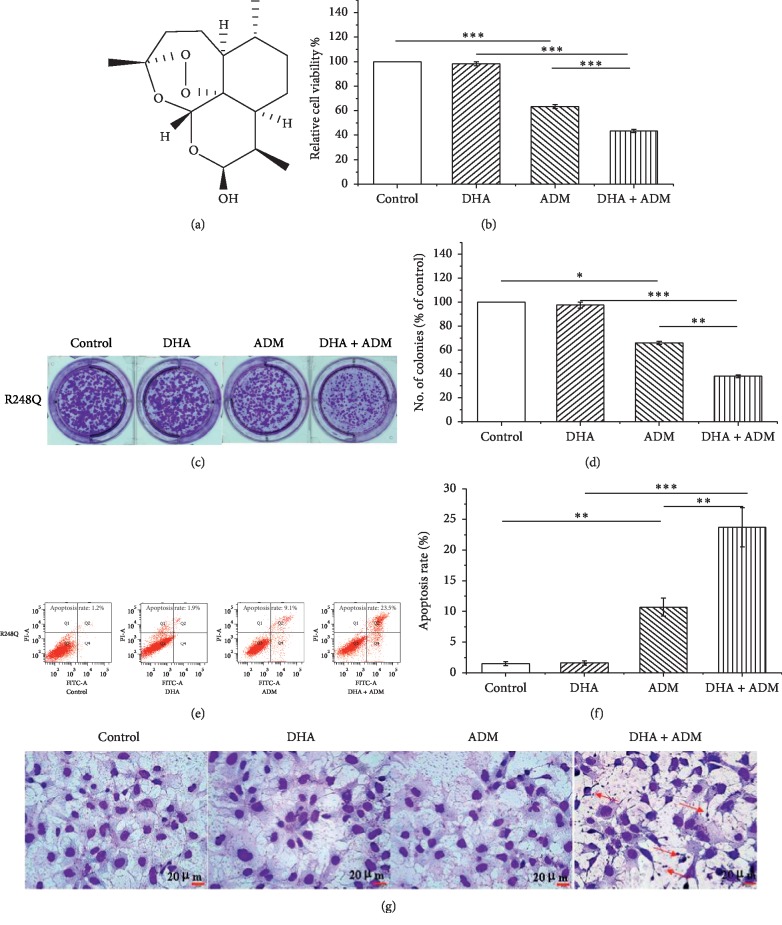
DHA sensitized p53 (R248Q)-expressing cells to ADM. (a) Chemical structure of DHA. (b) Detection of cell viability upon DHA combined with ADM by MTT assay. (c) Representative image of colony formation. (d) Quantitative graph of colony number. (e) Detection of cell apoptosis by flow cytometry. (f) Quantitative graph of apoptotic rates. (g) Morphological changes of cells (magnification ×400). The red arrowhead indicated membrane invagination, nuclear condensation, and vacolation in apoptotic cells. The data represent the mean ± SD of 3 independent experiments. ^*∗*^*p<0*.05, ^*∗∗*^*p* < 0.01, and ^*∗∗∗*^*p* < 0.001.

Since the combination of DHA with chemotherapeutic agents exerts synergistic effects in the treatment of many kinds of cancers [24, 25], we next explored whether DHA could enhance the sensitivity to ADM in p53 (R248Q)-expressing HCC cells. The chemical structure of DHA was shown in Figure 2(a) [26]. We treated p53 (R248Q)-expressing cells with combination of 5 *μ*M DHA and 2 *μ*M ADM and performed MTT assays, colony formation assays, and cell apoptosis analysis. MTT assays showed that ADM in combination with DHA exhibited significantly elevated inhibitory effects on cell viability compared with ADM treatment alone (Figure 2(b)). Colony formation assays showed that combination of ADM and DHA significantly decreased clone numbers compared with ADM treatment alone (Figures 2(c) and 2(d)). Furthermore, ADM in combination with DHA enhanced ADM-induced cell apoptosis (Figure 2(e)). There was significant difference in ADM-induced apoptosis between the combination group and ADM group (Figure 2(f)). Microscopic observation revealed that the apoptotic phenotype of the ADM and DHA combination group was more obvious than that of ADM treatment alone (Figure 2(g)). These data indicate that DHA synergistically enhances apoptosis-inducing effect of ADM in p53 (R248Q)-expressing HCC cells.

### 3.3. DHA Inhibits P-Gp Expression via Regulating the p53 (R248Q)-ERK1/2-NF-*κ*B Signaling Pathway

**Figure 3:**
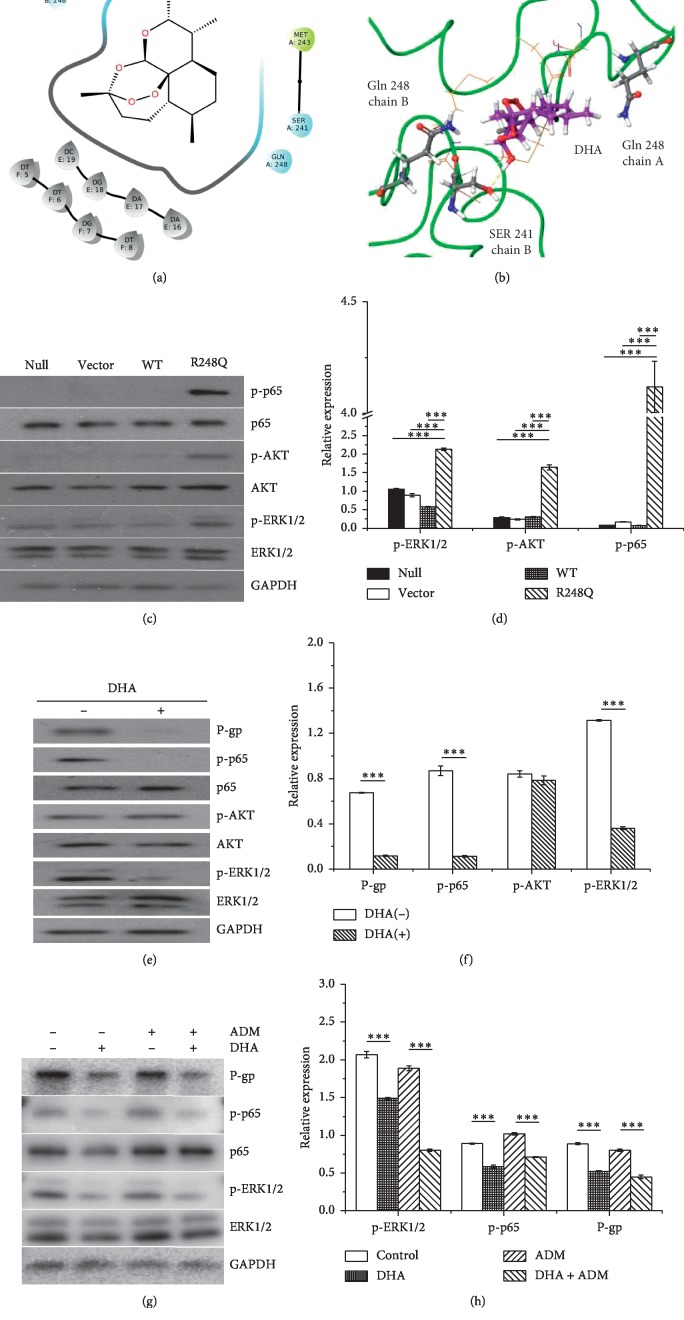
DHA inhibited P-gp expression via decreasing phosphorylation levels of ERK1/2 and p65 in p53 (R248Q)-expressing Hep3B cells. (a) The combining schematic diagram between DHA and p53 (R248Q) protein. (b) The 3D model of combining schematic diagram between DHA and p53 (R248Q) protein. (c) Representative image and (d) quantitative analysis of basal phosphorylation levels of AKT, ERK1/2, and p65 in Hep3B-derived clones by Western blot. (e) Representative image and (f) quantitative analysis of influence of DHA on P-gp expression in p53 (R248Q)-expressing cells detected by western blot. (g) Representative image and (h) quantitative analysis of influence of DHA on P-gp expression in ADM-treated p53 (R248Q)-expressing cells detected by western blot. The data represent the mean ± SD of 3 independent experiments. ^*∗∗∗*^*p* < 0.001.

We further studied the binding potential of DHA with mutant p53 (R248Q) protein. As shown in Figure 3(a), DHA bound to the middle of the two chains of p53 (R248Q), where the main amino acids interacting with DHA were Asn239, Ser241, Cys242, Met243, and Gln248. Besides, DHA could form hydrogen bonds with Ser241 in the B chain of p53 (R248Q) protein. The 3D model also showed that the main active site of drug-binding pocket of mutant p53 (R248Q) protein was Ser241 in the B chain, which formed a spatial conformation for DHA binding (Figure 3(b)). MM/GBSA predicted that the free energy of binding between DHA and mutant p53 (R248Q) was 10.58 kcal/mol, indicating that DHA has the potential to bind to mutant p53 (R248Q) protein.

Furthermore, western blot analysis showed that AKT, ERK1/2, and p65 were highly phosphorylated in R248Q but not in control cells or WT p53-expressing Hep3B cells (Figures 3(c) and 3(d)). However, DHA decreased P-gp expression and inhibited phosphorylation levels of both ERK1/2 and p65 but not AKT in p53 (R248Q)-expressing cells (Figures 3(e) and 3(f)). Furthermore, DHA decreased P-gp expression and phosphorylation levels of both ERK1/2 and p65 in ADM-treated p53 (R248Q)-expressing Hep3B cells (Figures 3(g) and 3(h)). These data suggest that DHA may inhibit P-gp expression via regulating the p53 (R248Q)-ERK1/2-NF-*κ*B signaling pathway.

### 3.4. DHA Decreases ADM Efflux in p53 (R248Q)-Expressing Hep3B Cells

**Figure 4:**
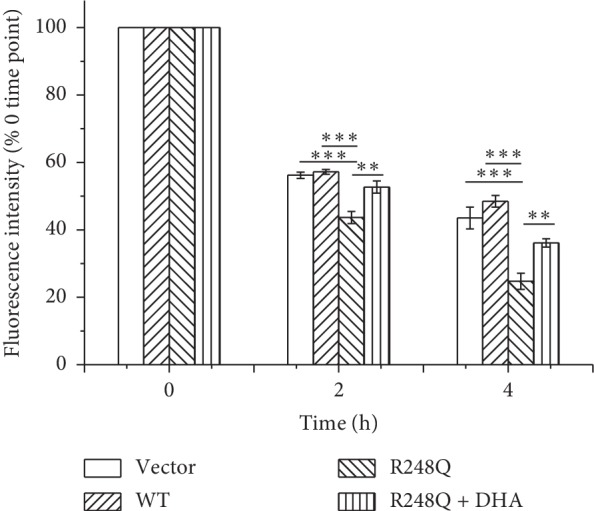
DHA decreased ADM efflux efficiency in p53 (R248Q)-expressing cells. Intracellular retention of ADM was detected by flow cytometry. The data represent the mean ± SD of 3 independent experiments. ^*∗∗∗*^*p* < 0.001 and ^*∗∗*^*p* < 0.01.

To further explore the mechanism underlying the enhancement effect of on ADM, the intracellular retention of ADM was examined by flow cytometry. As shown in Figure 4, p53 (R248Q)-expressing cells exhibited significantly decreased retention of ADM in comparison with either control cells or WT p53-expressing cells. However, DHA treatment significantly enhanced intracellular ADM retention in p53 (R248Q)-expressing cells. These results demonstrate that DHA decreases ADM efflux in p53 (R248Q)-expressing Hep3B cells.

## 4. Discussion

DHA, the major active product of Chinese antimalarial herb, has been reported to exhibit anticancer activity in various hepatoma cell lines [27–29]. Meanwhile, DHA is also a potential chemosensitizer and can synergistically enhance the effects of chemotherapeutics in many cancers [30]. However, the potential effect of DHA on reducing drug resistance of HCC cells harboring p53 mutant is not fully understood. In this study, we found that DHA could synergistically enhance ADM effects in the treatment of mutant p53 (R248Q)-expressing HCC cells. We further found that DHA could decrease P-gp expression via inhibiting p53 (R248Q)-activated ERK1/2 and NF-*κ*B signals, finally causing chemosensitivity in mutant p53 (R248Q)-expressing HCC cells. Our study indicates that DHA has the potential to serve as a chemosensitizer for drug-resistant HCC treatment.

p53 (R248Q), one of the most frequent DBD mutations of p53 protein, is able to induce P-gp expression, thus leading to resistance to chemotherapeutics in many human cancers [18,31–33]. Therefore, we introduced p53 (R248Q) mutation into p53-null HCC cells to construct the drug-resistant HCC clones. To avoid the heterogeneity caused by different positive clones, we mixed all the p53 (R248Q)-expressing clones of stable transfection together for the subsequent experiments. Our results showed that p53 (R248Q) greatly induced expression of P-gp. As a result, Hep3B cells expressing p53 (R248Q) exhibited significant resistance to ADM. This is consistent with a previous study that p53 (R248Q) induced ADM resistance in HCC cells [18]. Interestingly, there were significant differences in apoptosis rate at the baseline between R248Q and WT p53-expressing cells when cells were not treated with ADM. This is probably due to the proapoptotic effect exerted by WT p53, which induced transcription of *P21* and *BAX* genes, finally leading to proapoptotic effect in ADM-untreated Hep3B cells [34].

DHA exhibits cytotoxic effects at higher concentration but has little cytotoxic effect at lower concentration on cancer cells [24]. To study the synergistic effect of DHA with ADM, we must screen proper concentration of DHA which has little cytotoxic effect on Hep3B cells. Therefore, we treated mutant p53 (R248Q)-expressing HCC cells with different concentration of DHA and found that a concentration below 10 *μ*M hardly affected cell viability (data not shown). Therefore, to avoid cytotoxicity of DHA, we used 5 *μ*M DHA to treat p53 (R248Q)-expressing cells in our study. We found that 5 *μ*M DHA hardly affected viability or induced apoptosis in p53 (R248Q)-expressing Hep3B cells. However, 5 *μ*M DHA exhibited strongly synergistic effect by promoting ADM-induced cell cytotoxicity and apoptosis in p53 (R248Q)-expressing cells. This is consistent with a previous study that DHA served as a synergistic agent to promote chemotherapeutics-induced apoptosis in NSCLC cells [35]. Above all, we concluded that DHA sensitized mutant p53 (R248Q)-expressing HCC cells to ADM.

We further explored the underlying mechanism by which DHA increased ADM sensitivity in mutant p53 (R248Q)-expressing HCC cells. p53 (R248Q) has the potential to stimulate PI3K-AKT, MAPK-ERK1/2, and NF-*κ*B signals, subsequently regulating the expression of cancer-related genes [36]. Furthermore, PI3K-AKT, MAPK, and NF-*κ*B signals could participate in the expression of P-gp [37–39]. So, we detected the phosphorylation level of AKT, ERK1/2, and p65 in each group. We found that mutant p53 (R248Q)-expressing cells exhibited highly phosphorylated AKT, ERK1/2, and p65, suggesting the possible role of these signals in regulating P-gp expression. DHA could inhibit tumor progression by interfering with MAPK signaling [40]. Meanwhile, DHA could interfere with P-gp function [41, 42]. Furthermore, DHA could synergistically enhance the anticancer effects of other chemotherapeutic agents by blocking transduction signals such as AKT-mTOR, JAK-STAT3, and NF-*κ*B [24, 25]. Therefore, we suppose that DHA could decrease the expression of P-gp by inhibiting phosphorylation of AKT, ERK1/2, or p65, which finally caused the sensitization of p53 (R248Q)-expressing HCC cells to ADM. To validate our hypothesis, we firstly simulated the docking model between p53 (R248Q) protein and DHA. We found that DHA had the potential of directly binding with Ser241 of p53 (R248Q) protein. Considering the stimulating effect of p53 (R248Q) protein on p-AKT, p-ERK1/2, and p-p65, we next detected the effect of DHA on the phosphorylation levels of AKT, ERK1/2, and p65. We found that DHA had remarkable inhibitory effects on phosphorylation levels of ERK1/2 and p65. Yet, DHA barely had inhibitory effect on phosphorylation level of AKT, suggesting that DHA could bind with p53 (R248Q) protein to decrease p53 (R248Q)-stimulated ERK1/2 and NF-*κ*B signals. Notably, DHA could prominently decrease P-gp expression in p53 (R248Q)-expressing Hep3B cells whether cells were treated with ADM or not. Taken together, we concluded that DHA decreased P-gp expression via binding with p53 (R248Q) protein, which in turn downregulated p53 (R248Q)-ERK1/2-NF-*κ*B signals in p53 (R248Q)-expressing HCC cells. P-gp is able to pump chemotherapeutic agents, such as ADM, from cytoplasm to extracellular environment, helping to enhance drug resistance in cancer cells [9, 43]. To further confirm the inhibitory effect of DHA on P-gp expression, we finally compared the ADM efflux efficacy between DHA-untreated and treated p53 (R248Q)-expressing cells and found that DHA greatly reduced P-gp-mediated ADM efflux in p53 (R248Q)-expressing cells.

## 5. Conclusions

In conclusion, DHA reduced P-gp expression in p53 (R248Q)-expressing HCC cells via downregulating the p53 (R248Q)-ERK1/2-NF-*κ*B signaling pathway, which finally reduced ADM resistance. Therefore, DHA may be a synergistic agent for ADM in the treatment of drug-resistant HCC induced by mutant p53 (R248Q).

## Data Availability

The data used to support the findings of this study are included within the article.
